# Acoustical structured illumination for super-resolution ultrasound imaging

**DOI:** 10.1038/s42003-017-0003-5

**Published:** 2018-01-22

**Authors:** Tali Ilovitsh, Asaf Ilovitsh, Josquin Foiret, Brett Z. Fite, Katherine W. Ferrara

**Affiliations:** Department of Biomedical Engineering, University of California, Davis, 95616 CA USA

## Abstract

Structured illumination microscopy is an optical method to increase the spatial resolution of wide-field fluorescence imaging beyond the diffraction limit by applying a spatially structured illumination light. Here, we extend this concept to facilitate super-resolution ultrasound imaging by manipulating the transmitted sound field to encode the high spatial frequencies into the observed image through aliasing. Post processing is applied to precisely shift the spectral components to their proper positions in *k*-space and effectively double the spatial resolution of the reconstructed image compared to one-way focusing. The method has broad application, including the detection of small lesions for early cancer diagnosis, improving the detection of the borders of organs and tumors, and enhancing visualization of vascular features. The method can be implemented with conventional ultrasound systems, without the need for additional components. The resulting image enhancement is demonstrated with both test objects and ex vivo rat metacarpals and phalanges.

## Abstract (web summary)

Tali Ilovitsh *et al.* describe a new method, acoustical structured illumination, for generating super-resolution ultrasound images. The method applies principles from structured illumination microscopy and can be adapted to existing ultrasound systems without the need for additional components.

## Introduction

Ultrasound is a safe, non-invasive, real-time, and cost effective clinical imaging modality that can be applied to image tissue deep within the body. As such, it is one of the most widely used imaging modalities for the detection and diagnosis of disease, in applications including fetal, genitourinal, cardiovascular, orthopedic, breast, and abdominal imaging^1^. Ultrasound is also applied as a screening tool for early cancer detection due to its ability to differentiate cysts from solid masses based on target size, shape, and relative echogenicity^2, 3^. Advances in ultrasound technologies have now led to user-programmable systems, capable of a nearly infinite variety of transmitted pulse trains, and schemes for image reconstruction. Despite these advances, ultrasound still suffers from limitations in resolution, contrast and signal to noise ratio (SNR), and from artifacts^4^. We report on a new technology to implement a structured illumination mode for ultrasound imaging with the specific goal of improving spatial resolution. The development of methods for super-resolution ultrasound imaging, in addition to currently available optical structured illumination microscopy (SIM), is significant as optical microscopy is limited in tissue penetration, whereas ultrasound systems are designed to image the deep organs. Safe, low cost, and non-invasive imaging deep within the body at microscopic scales remains an important goal in biomedical imaging. For many clinical applications, it would be desirable to resolve structures with sub-diffraction dimensions^5^.

The spatial resolution of an optical or ultrasound imaging system is limited by diffraction to length scales of approximately half of the wavelength of the transmitted beam. Any object that is significantly smaller than the spatial resolution of the system can be considered as a point scatterer and appears to be blurred in the resulting image, where the observed shape is determined by the point spread function of the system. Two or more point reflectors spaced more closely than the resolution limit cannot be distinguished. In recent years, optical super-resolution microscopy techniques have revolutionized the observation of living structures at the cellular scale, offering an improved spatial resolution as compared to the traditional diffraction limit^6, 7^. Time multiplexing super-resolution^8, 9^, more commonly known as SIM^10^, exceeds the diffraction limit by illuminating the sample with a series of known patterns, which cause normally inaccessible high-resolution information to be encoded into the observed image. The method is based on the moiré effect; when two patterns that contain frequencies above the cutoff frequency of the system are superimposed one on top of another, a beat pattern will appear in their product at a frequency designed to be below the cutoff of the system. This pattern can be captured by the receiver and used to recover the unknown high-resolution data, corresponding to a synthetic increase in the effective transducer aperture without a change in the aperture’s physical dimensions. Typical periodic illumination patterns include multifoci^11, 12^ and sinusoidal striped patterns^10^. As the patterns are generated by the imaging system, they are limited by its cutoff frequency. Therefore, the effective resolution can be doubled compared to the default one-way focusing resolution; moreover, a further increase in resolution can be achieved using nonlinear SIM^13^.

We propose using an acoustical structured illumination (ASI) in which the patterns can be generated and captured by the transducer itself. In ASI, the patterns result from the acoustic wavefront manipulation. This is achieved by controlling the phase and apodization of each individual transducer element, providing the method with the distinct advantage of being dynamic and reconfigured in real-time. In ultrasound, multi-foci patterns have been used previously to generate a uniform temperature field for hyperthermia treatments^14–16^, for ultrasonic neuro-modulation^17^, for the generation of acoustic holograms^18, 19^, and for multiline cardiac imaging^20, 21^. To our knowledge, they have not been used for ultrasound super-resolution imaging. The patterns to be generated can be calculated by many distinct methods. As most multi-foci techniques have been applied for therapeutic purposes, they have been based on continuous wave insonation. The continuous wave algorithms include the conjugate field method^14^, the pseudo-inverse method^15^, and the Gerchberg–Saxton phase retrieval algorithm^22^. Among these methods, the Gerchberg–Saxton algorithm yields the best results in efficiency and focal spot uniformity^17^. By comparison, the multiline transmission method (MLT)^21, 23^ is a pulse echo imaging method that generates multiple foci by coherently summing the phases of individual foci. Here, we use a modified Gerchberg–Saxton algorithm in a pulse echo acquisition and compare the pattern to that generated by MLT. Other ultrasound techniques geared to improve spatial resolution include: ultrasound deconvolution^24, 25^, monostatic synthetic aperture^26^, and constrained least squares beamforming^27^. These methods have drawbacks for real-time medical applications, including the enhancement of both the signal and the noise, which reduces the SNR, the introduction of artifacts, and poor contrast resolution due to higher side lobes.

**Fig. 1:**
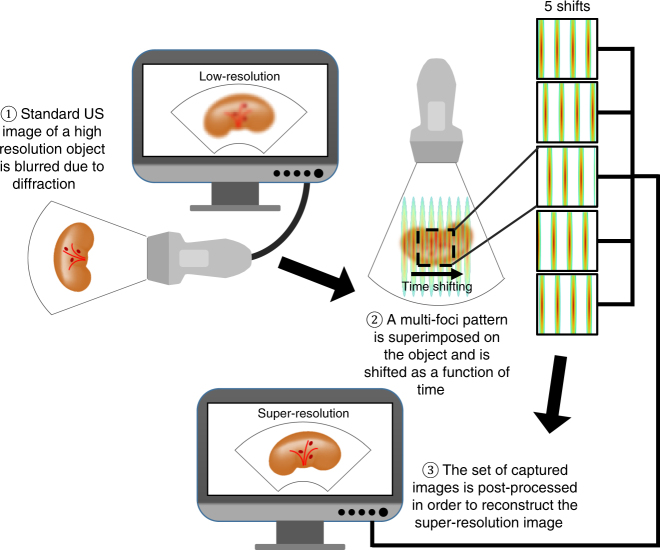
Schematic illustration of ASI steps of operation (left to right). An object that contains sub-diffraction features is imaged with ultrasound. A multifocal pattern is generated at the position of the object and the echoes are captured by the transducer. Five emitted fields, corresponding to five shifts of the pattern, are transmitted sequentially, and a set of five images is captured. The set of captured images is post processed to reconstruct the super-resolution image, where sub-diffraction features are visible in comparison to the original low-resolution image

The method described here allows for real-time image acquisition. It requires a shift of the emitted pattern between subsequent pulses, followed by the acquisition of the images, where all of the phase and apodization maps are calculated before the imaging session. During an experiment, the emitted field can be changed dynamically to generate shifted patterns, acquire the images and reconstruct the super-resolution image (Fig. 1). Similar to SIM^9, 28, 29^, the reconstruction process includes the multiplication of each captured ultrasound image by its corresponding decoding pattern, summing the products and averaging the results. Although we provide examples here of high dynamic range in vitro and ex vivo targets, the methods can be extended to in vivo imaging in the future.

## Results

### Theoretical background

**Fig. 2:**
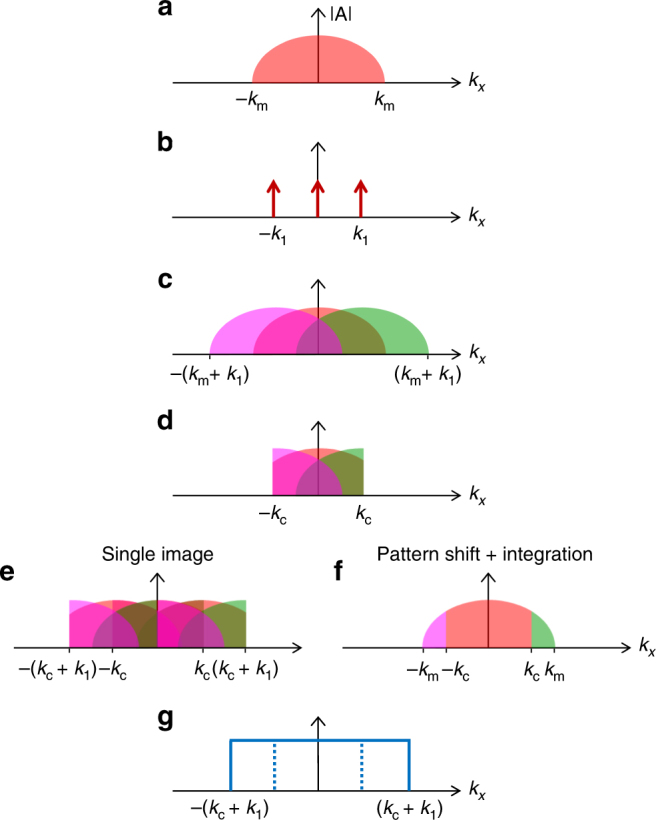
Schematic illustration of structured illumination in *k*-space. **a** The imaged object has spectral components ranging from −*k*_m_ to *k*_m_. **b** The Fourier transform of the pattern generated in ASI at the focal depth. The pattern has three non-zero components located at −*k*_1_, 0, *k*_1_. **c** Superposition of the pattern on the object in the spatial domain, translates into a convolution between their *k*-space components. Because of the frequency mixing, duplications of the original object occur. **d** On receive, the duplications of the original object in *k*-space are multiplied by the rectangular receive transfer that has a cutoff frequency of *k*_c_. As a result, the result in **c** is low-pass filtered; however, the product contains the high-resolution data downshifted due to the additional duplications. **e** Post processing includes the multiplication of the result by a second pattern, similar to the ideal transmitted pattern, generating additional duplications. Using a single image, the frequency components are not fully separated; thus, artifacts appear in the reconstructed image. **f** A precise reconstruction of the object is created by shifting the pattern, multiplying each captured image by a corresponding decoding pattern, and summing the product images. **g** The equivalent lateral transfer function of ASI is rectangular with a width ranging from -(*k*_c_ + *k*_1_) to +(*k*_c_ + *k*_1_)

Our goal is to improve the lateral resolution of ultrasound imaging systems. Ultrasound lateral spatial resolution is limited to length scales set by the Rayleigh criterion according to:

1 $${\mathrm{FWHM = }}{\mathrm const} \times \lambda \frac{z}{D},$$

where FWHM is the full width at half maximum of the beam width, *λ* is the wavelength corresponding to the center frequency of the transmitted beam, *z* is the distance between the transducer and the focal depth, *D* is the transducer aperture size and const is a parameter that is determined by the transducer apodization. For a rectangular aperture, const = 1.206^30^. For two-way focusing, ultrasound beams are swept through a region by weighting and delaying the pulses transmitted to and received from each element of a transducer appropriately. In this work, one-way focusing is achieved by transmitting a planar wave that illuminates the entire field and focusing on reception. The axial resolution is limited by the duration of the applied pulse and, for a single-cycle pulse, can be as small as half the acoustic wavelength when imaging in reflection^31^. Our method (summarized in Fig. 2) improves lateral resolution while maintaining excellent axial resolution through the use of single-cycle transmission.

Let us assume that the imaged object has spectral components ranging from −*k*_m_ to *k*_m_ (Fig. 2a). Beam formation by an ultrasound transducer introduces a low-pass filter for lateral spatial frequencies. For a flat apodization (all elements are weighted equally), in the harmonic regime, the frequency space (*k*-space) representation of the beam formation process for a rectangular aperture is rectangular in shape on either transmission or reception with a cutoff frequency of *k*_c _^32^ (Supplementary Fig. 1). Although a pulsed excitation yields an error function as the *k*-space lateral transfer function, as in ref. ^33^, for simplicity, we assume a rectangular *k*-space transfer function. In two-way focused ultrasound, the two-way lateral transfer function is equal to the convolution between the transfer function on transmission or reception. Assuming both are rectangles with the same width, the two-way transfer function is a triangle with twice the cutoff frequency as compared to the one-way focusing^34^. As such, the spatial resolution of the resulting received image is higher; however, in two-way focusing high frequencies remain attenuated. Modern programmable ultrasound can also perform high-speed imaging by transmitting a plane wave and creating a parallel receive beamforming only on receive. For plane wave imaging, the Fourier transform of the object is multiplied by the rectangular lateral transfer function on receive only and thus frequencies above the cutoff frequency cannot be observed (Supplementary Fig. 1).

In ASI, a lateral multifocal pattern is generated at a pre-determined depth with a constant distance Δ*d* between each pair of foci, thus the spatial frequency *k*_1_ of this periodic pattern is the inverse of Δ*d*, i.e., *k*_1_ = 1/Δ*d*. In *k*-space, the pattern is an infinite series of delta functions, multiplied by the lateral transfer function. After this multiplication, three delta components remain and as such the pattern has three non-zero *k*-space frequencies with one at the origin due to the DC component, and two components offset symmetrically from the origin by a distance determined by the frequency of the pattern, *k*_1_ (Fig. 2b). In the spatial domain, an infinite raised cosine function is an example of a grating pattern resulting from single-cycle transmission from an infinite transducer; however, with a finite transducer aperture, a laterally windowed cosine pattern is transmitted, resulting in three narrow sinc functions in *k*-space, rather than delta functions. For simplicity, here we will assume an infinite raised cosine pattern. When we superimpose the pattern at the object’s position, the resulting scattered wave from the object is the product of the insonation pattern and the scattering function of the object. In *k*-space, this multiplication becomes a convolution resulting in a mixing of the frequencies of the original data and the pattern. As a result, two additional duplications of the k-space information will be generated, each offset to be centered around ±*k*_1_ (Fig. 2c).

The echoes undergo low-pass filtering during the receive beam-formation process, which imposes a *k*-space rectangular low-pass filter with a cutoff frequency of *k*_c_. The received echoes also contain high-frequency components of the original object that have been downshifted due to the encoding process (Fig. 2d). The super-resolution image is reconstructed using computer post processing, which restores the downshifted frequencies by multiplying each received image by an idealized pattern with the same *k*-space frequencies of 0, ±*k*_1_^8^ (Fig. 2b) (see mathematical framework in Supplementary Note 1). In *k*-space, after this multiplication, two additional duplications are generated. When capturing only one image, the result in Fig. 2e is composed of the sum of the three contributions, and thus artifacts that stem from undesired duplications are included in the result. Therefore, a perfect super-resolution image cannot be assembled from a single image, as the frequency components are not separable. To accurately reconstruct the object, a set of images are acquired where the pattern is shifted between subsequent pulses. The shifts are chosen such that the scan covers a full period of the pattern, Δ*d*. Typically, three shifts are used to maximize acquisition speed, or five shifts are chosen to increase super-resolution image quality^10^. A spatial shift of the pattern, translates into an addition of a phase component for each of these shifts.

Let us index the *k*-space components –*k*_1_, 0, *k*_1_ of the encoding grating as *m* = −1, 0, +1 and for the decoding grating *n* = −1, 0, +1, respectively. After the initial multiplication with a shifted pattern, the phase term for each shift is $e^{2\pi ik_1\phi m}$, where *m* is the index of the shift and *ϕ* is the size of the shift. In the reconstruction process, the obtained result is multiplied by the decoding grating that is shifted similarly to the encoding grating, such that the added phase term is $e^{2\pi ik_1\phi n}$, where *n* is the index of the shift. The total added phase to each component is the multiplication of these phase terms, resulting in $e^{2\pi ik_1\phi (m + n)}$. Next, the product is integrated over one period of the pattern, and since only the phase term is changed between pulses:

2 $$\frac{1}{{{\mathrm{\Delta }}d}}{\int}_{{- {\mathrm{\Delta }}d/2}}^{{{\mathrm{\Delta }}d/2}} { \ldots e^{2\pi if_1\phi (m + n)}d\phi {\mathrm{ = }}\left\{ {\begin{array}{*{20}{c}} {1,\quad m{\mathrm{ + }}n{\mathrm{ = }}0} \\ {0,\quad m{\mathrm{ + }}n \ne 0} \end{array}} \right.} .$$

This result indicates that only specific terms of the frequency mixing (where *n* = -*m*) appear after the integration, whereas all other terms, which are the undesirable artifacts, are canceled. The method is capable of transmitting frequencies up to *k*_c_ + *k*_1_, thus for *k*_m_ < *k*_c_ + *k*_1_ the object can be accurately reconstructed (Fig. 2f). In addition, the transmitted pattern itself is limited by the cutoff frequency of the system, and therefore *k*_1_ ≤ *k*_c_. The resulting lateral transfer function is composed of the sum of three rectangular transfer functions and to achieve a flat effective transfer function, a low-resolution image is subtracted from the reconstructed image. Here, the low-resolution image is the average of the five captured ASI images. The lateral transfer function for ASI is therefore a rectangle with a cutoff at *k*_c_ + *k*_1_ (Fig. 2g). For the case where *k*_1_ = *k*_c_, the transfer function is a rectangle with a width of 2*k*_c_. This provides both a uniform contrast and high resolution, while doubling the transfer function of plane wave imaging.

### Design and validation of patterns for wide-band ASI

**Fig. 3:**
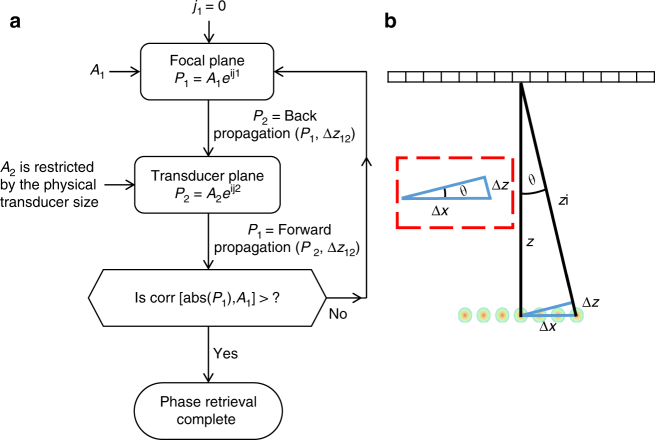
Design of the focal pattern, including constraints imposed on the number of foci. **a** Flowchart of the modified Gerchberg–Saxton process designed to create the pressure *P*_1_ at the focus based on the pressure *P*_2_ on the transducer surface. Initially, the desired focal pattern amplitude, *A*_1,_ and its distance *z* from the transducer are defined and a zero phase *φ*_1_ is imposed. This field is back propagated to the transducer plane using the angular spectrum method. The result is the amplitude *A*_2_ and phase *φ*_2_ that define the second plane *P*_2_. An iterative process continues until the correlation coefficient between *A*_1_, and the desired amplitude, is higher than a pre-determined threshold. **b** Geometric model for the temporal and spatial interference requirement

The Gerchberg–Saxton algorithm is an iterative algorithm for retrieving the phase of a propagating field from a pair of imaging planes related via a propagating function. In ultrasound, the angular spectrum is used for near-field propagation with low computational cost^35^. The phase and apodization distributions of a transducer are computed by iteratively propagating the acoustic wave backward and forward from the image plane to the transducer until the algorithm converges based on a pre-determined threshold. Let *P*(*x*, *z*) denote the complex harmonic pressure at a single frequency in a uniform medium. The acoustic field is expressed as:

3 $$P\left( {x,z} \right){\mathrm{ = }}A\left( {x,z} \right)e^{j\varphi \left( {x,\,z} \right)},$$

where *A* and *φ* are the amplitude and phase terms, respectively, and *P*_1_ is the pressure at the focus, whereas *P*_2_ is the pressure at the transducer surface. Initially, the amplitude in the focal plane, *A*_1_ and its distance Δ*z*_12_ from the transducer is defined and a zero phase *φ*_1_ is imposed. This field is back propagated to the transducer plane using the angular spectrum method. The result is the amplitude and phase that define *P*_2_. The amplitude of *A*_2_ is set to zero outside the aperture of the transducer, based on its physical dimensions, whereas the calculated phase *φ*_2_ is maintained. *P*_2_ is forward propagated by another Δ*z*_12_ to *P*_1_, where the desired amplitude of *A*_1_ is imposed, whereas the calculated phase *φ*_1_ is retained. This process is iterated until the correlation coefficient between *A*_1_, calculated with forward propagation, and the absolute value of *P*_1_ is higher than a pre-determined threshold. The process requires a few tens of iteration cycles to converge. A flowchart of the method is presented in Fig. 3a.

The above result assumes a time dependence of harmonic vibration. In the case of ultrasound imaging, a single cycle of sound is transmitted and therefore the dynamic range between the focal locations and background is reduced.

The number and spacing of foci determines the size of the region of interest (ROI) at the focal depth. A larger number of foci will facilitate imaging a larger ROI; however, the transmitted energy will be divided equally between all foci, therefore the SNR will decrease if the time averaged energy is constrained. An additional limitation on the number of foci stems from the axial length of the transmitted pulse. Temporal and spatial interference of all transmitted cycles must occur to generate multiple foci simultaneously. With continuous wave transmission, any desired number of foci can be generated. However, in pulse echo imaging, where the ideal pulse length is 1 cycle, a restriction on the width of the pattern and the number of foci is imposed. In Fig. 3b, *Δz* describes the axial extent of the pattern, where

4 $${\it{\Delta }}z{\mathrm{ = }}z - z_{\mathrm{i}}.$$

*z* is the vertical distance of the pattern, and *z*_i_ is the distance to the most remote foci. Temporal and spatial pulse interference occurs when Δ*z* < *L* and *L* is the length of the pulse. From the triangle similarity between the two right triangles (black and blue), and by assuming a small angle approximation:

5 $$\frac{{{\it{\Delta }}z}}{{{\it{\Delta }}x}} \cong \frac{{{\it{\Delta }}x}}{z}.$$

Δ*x* is approximately equal to half of the width of the pattern. Isolating Δ*x*:

6 $${\it{\Delta }}x \cong \sqrt {z \times {\it{\Delta }}z} {\mathrm{ < }}\sqrt {z \times L} \mathop { \to }\limits^{1\,{\mathrm{cycle}}} \sqrt {z \times \lambda } .$$

**Fig. 4:**
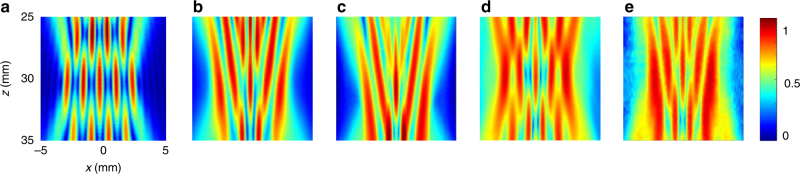
Normalized multifocal pattern measurement displayed in a linear scale. Axes and colorbar are common to all subfigures. **a** Simulated emitted ultrasound field for continuous wave transmission, using the modified Gerchberg–Saxton algorithm for Δ*d* = 1.1 mm. **b** Simulated emitted ultrasound field for 1 cycle transmission, using the modified Gerchberg–Saxton algorithm for Δ*d* = 1.1 mm. **c** Experimental hydrophone measurement of the pattern presented in **b**. **d** Simulated emitted field for 1 cycle transmission using the MLT method for Δ*d* = 1.1 mm. **e** Experimental hydrophone measurement of the pattern presented in **d**

Scanning a larger ROI can be accomplished by combining multiple lateral and axial scans at intervals of 2Δ*x*. Alternatively, Δ*x* can be increased by imaging objects at deeper depth, or by transmitting longer waveforms. For imaging parameters of 1 cycle at a center frequency of 3.6 MHz and a pattern with Δ*d* = 1.1 mm at a depth of *z* = 30 mm in water (speed of sound 1490 m s^−1^), Δ*x* < 2.8 mm, the full pattern width is 2Δ*x* and *k*_c_ = 1.8 cycles mm^−1^. Choosing Δ*d* = 1.1 mm (0.5/*k*_c_) limits the number of foci to 5. Figure 4 presents an example of a pattern of five foci with the given imaging parameters. Simulated emitted fields for continuous wave and 1 cycle transmission are presented in Fig. 4a and 4b, where the effective dynamic range is 45.4 and 30.8 dB, respectively. The experimental field corresponding to the 1 cycle transmission  was recorded with a calibrated wide-band needle hydrophone (Fig. 4c) and the measured dynamic range was 27 dB indicating the similarity between the experimental and simulated fields. Although the MLT method can also generate multiple foci (Fig. 4d), the foci separation and uniformity are coarse with comparison to the modified Gerchberg–Saxton algorithm, and the simulated dynamic range was 15 dB. The experimental field corresponding to MLT transmissions was also recorded with a calibrated wide-band needle hydrophone (Fig. 4e), yielding a dynamic range of 13.9 dB. Owing to the superior performance, the algorithm used in the experiments was the modified Gerchberg–Saxton algorithm. The cross sections along the focal depth for Fig. 4a–h are presented in Supplementary Fig. 2.

The achievable improvement in resolution of ASI is governed by the frequency of the generated pattern, where the pattern spatial frequency is inverse to the pattern spacing *k*_1_ = 1/Δ*d* and the resolution enhancement factor equals (*k*_1_ + *k*_c_)/*k*_c_. To achieve the maximum spatial frequency cutoff of 2*k*_c_, the spatial frequency of the pattern must equal the cutoff frequency and as a result Δ*d* must be similar to the diffraction limit. As such, tradeoffs exist between resolution, ROI size and SNR. Higher resolution requires decreased foci spacing, which reduces the size of the ROI; alternatively, more foci can be generated at the cost of a reduction in the SNR for a constant transmitted power. To provide examples of the tradeoffs for the foci spacing, Supplementary Fig. 3 presents the line profile at the focal depth for a five-foci pattern with Δ*d* values of 0.8 (0.7/*k*_c_), 0.58 (0.97/*k*_c_), and 0.55 (1/*k*_c_) mm. As expected, the contrast of the generated pattern is reduced as Δ*d* decreases, until Δ*d* drops below the diffraction limit and the pattern cannot be resolved. For the pattern spacings presented in Fig. 4 (Δ*d = *1.1 mm) and in Supplementary Fig. 3 as above, the resolution enhancement as compared to one-way focusing is 1.5, 1.7, 1.97, and 2, respectively.

### Experimental validation of ASI

**Fig. 5:**
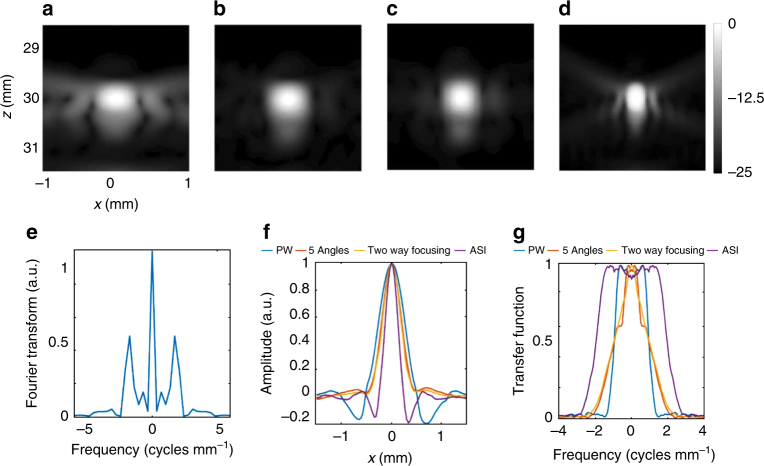
Experimental results based on imaging of a wire target with: **a** plane wave imaging with one-way focusing. **b** Five-angle coherent compounding. **c** Two-way focusing. **d** ASI method. **a**–**d** Log-scaled magnitude images presented with −25 dB dynamic range. Axes are common to **a**–**d**. **e** Fourier transform of the focal depth line profile of the ASI pattern presented in Supplementary Fig. 3. **f** Pressure field-line profiles at the focal depth of 30 mm for **a**–**d**. **g** Fourier transform (*k*-space representation) of **e**

The shape of the transfer function for the different imaging methods was validated using a point target experiment (Fig. 5). The target was a 0.1 mm copper wire that was positioned in a water tank at a depth of 30 mm. Initially, the wire was imaged using three standard ultrasound imaging methods: plane wave transmission with parallel receive beamforming, coherent compounding of five-angled plane wave, and two-way focusing (Fig. 5a–c, respectively). For ASI, the patterns were generated with Δ*d* = 0.58 mm (a single-emitted pattern is presented in Supplementary Fig. 3b), where the spacing was chosen to be near to the diffraction limit to achieve the maximal resolution enhancement using the method. The ASI result (Fig. 5d) match the theoretical background predictions in that the lateral width of the wire-point spread function is minimized using the ASI technique. Figure 5e presents the Fourier transform of the focal depth line profile of the ASI pattern presented in Supplementary Fig. 3b. Figure 5f shows the pressure field line profiles at the focal depth of 30 mm for Fig. 5a–d. The *k*-space representation which is the Fourier transform of Fig. 5f is shown to approximate the expected rectangle (as an approximation to an error function) with a FWHM width of the transfer function of ASI of ~4 cycles mm^−1^, exceeding that of the other techniques (Fig. 5g).

**Fig. 6:**
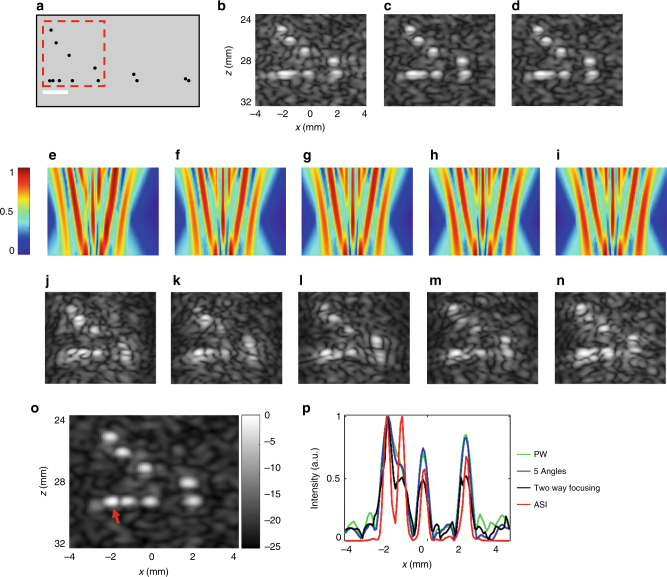
Experimental results with a multipurpose ultrasound imaging phantom. Results are presented in 25 dB dynamic range log scale. Axes are common to **b**–**n**. **a** Lateral and axial resolution targets. The lateral target was located at depths of 30 mm. The target is composed of 0.8 mm nylon wires, with lateral and axial separation distances in mm of 0.25, 0.5, 1, 2, 3, and 4. White scale bar is 2 mm. **b** Plane wave reference image. **c** The result of coherently compounding five plane wave reference images. **d** Two-way focused reference image. **e**–**i** Five emitted pressure fields. The generated pattern is shifted by 0.22 mm between successive images. **j**–**n** Corresponding echoed images. **o** Super-resolution reconstructed image. **p** Cross section along the lateral resolution target (*z* = 29.3 mm), for the reference images **b**–**d** and the super-resolution image **o**. Example selected from *n* = 3 repetitions

Further experiments were performed with an ultrasound resolution target (Fig. 6a) where the depth of the target was 29 mm and the diffraction-limited resolution was therefore 0.56 mm based on Eq. 1. Thus, wires separated by 0.50 mm would not be resolvable. For this case, since the required resolution enhancement was smaller than two, we increased the size of the ROI by choosing a spacing of 1.1 mm between the foci. The pattern was designed for this depth with five equally distributed foci. Five phase and apodization maps, corresponding to five shifts of the pattern, were calculated using the modified Gerchberg–Saxton algorithm, where the shift between successive frames equals one-fifth of the pattern’s period, i.e., 0.22 mm. For comparison, we first acquired a set of standard low-resolution reference images, of the same scene, including the following methods: one-way focused imaging (plane wave transmission) (Fig. 6b), coherently compounding five-angled plane wave acquisitions (Fig. 6c), and two-way focused imaging (Fig. 6d). The super-resolution imaging process included the successive emission of the five shifted patterns, and the simulated emitted fields are shown in Fig. 6e–i. Five echoed fields were captured and used for the post processing reconstruction of the super-resolution image; the magnitude images of the corresponding echoes are provided in Fig. 6j–n. Post processing utilized decoding patterns that were identical to the emitted patterns (Fig. 6e–i). Next, each captured image was multiplied by its corresponding decoding pattern and the result was averaged. A low-resolution image that is composed of the average of the five captured ASI images was subtracted from the result. An optional step that increases the contrast of the super-resolution image and reduces artifacts is to multiply the final image by a single low-resolution reference image, such as Fig. 6b. Following this step, the final super-resolution image is presented in Fig. 6o, and Fig. 6p is a cross section in the center of the lateral resolution target (*z* = 29.3 mm). The cross section demonstrates that the super-resolution image improves the target resolution. For the three other methods, the resolution and contrast were not sufficient to resolve wires spaced by a distance <0.5 mm, whereas in the super-resolution image the wires are resolved. The left pair of wires (red arrow in Fig. 6o) demonstrates the limitations of the technique as two wires separated by 0.25 mm remain diffraction-limited and cannot be resolved. A comparison of the performance of the blind deconvolution method, which was performed based on ref. ^24,^ is presented in Supplementary Fig. 4. The results indicate that under realistic imaging conditions, the resolution improvement is less than that achieved in the ASI method.

**Fig. 7:**
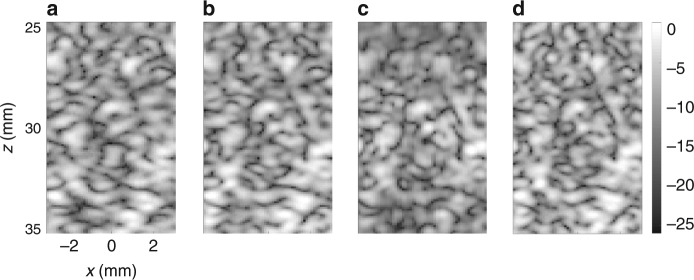
Experimental measurements of the background speckle in a homogeneous multipurpose ultrasound imaging phantom. Axes are common to **a**–**d**. Images are presented with a dynamic range of 25 dB for an image acquired with: **a** one-way focusing. **b** Coherent compounding of five-angled plane waves. **c** Two-way focusing, and **d** ASI imaging. Example selected from *n* = 3 repetitions

A speckle-generating target was then imaged within the same phantom, the resulting image was log compressed (Fig. 7) and the image autocorrelation function was calculated^36, 37^. For plane wave imaging, five-angle coherent compounding, two-way focusing and ASI (Fig. 7a–d, respectively), the main lobe FWHM was 0.6, 0.52, 0.51, and 0.37 mm, respectively. The autocorrelation FWHM value represents the size of the speckle resolution cell, and its reduction in ASI indicates an advantage of the method.

Images were also acquired from a 2 mm diameter cyst phantom at a depth of 45 mm (Supplementary Fig. 5) using both the conventional US methods and the ASI method. The contrast, contrast to noise ratio, and speckle resolution cell size were calculated according to ref. ^38^ and Supplementary Note 2. As expected, the contrast and contrast to noise ratio values are similar for ASI and two-way focusing (Supplementary Table 1) and superior to the other methods, and the speckle dimensions are smaller for ASI due to the enhanced resolution.

**Fig. 8:**
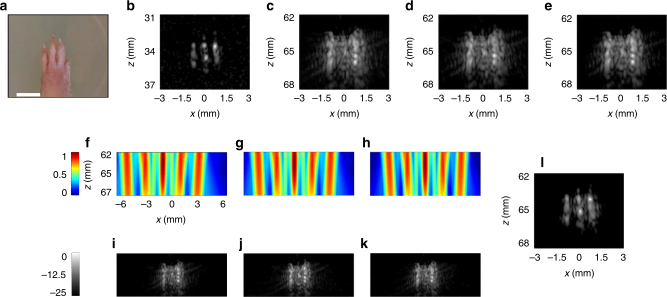
Experimental results obtained by imaging an ex vivo paw from a Sprague–Dawley rat. Results are presented in 25 dB dynamic range log scale with jet and gray colorbars common to all subfigures. Axes are common to **f**–**k**. **a** An image of the sample. The ultrasound images were acquired along the phalanges distal to 4th phalange. White scale bar represents 3 mm. **b** High-resolution image captured at *z* = 34 mm, where the phalanges are resolvable. For all other images, the position of the paw was shifted to 65 mm. **c** Plane wave image. **d** The result of coherently compounding plane wave images acquired from five angles. **e** Two-way focused image. **f**–**h** Three predicted emitted pressure fields. **i**–**k** Corresponding images. **l** Super-resolution reconstructed image. Example selected from *n* = 3 repetitions

In the second experiment, ex vivo phalanges within the rat paw (Fig. 8a) were imaged along the axis of the phalange. This cross section contained three phalanges that were spaced by ~0.8 mm. Initially, the paw was positioned at a depth of ~34 mm inside the agarose and for this distance, the diffraction limit was 0.61 mm; thus, the three phalanges were resolvable and the image captured at this depth was used for validation of the final super-resolution result (Fig. 8b). A spacer of agarose was inserted between the transducer and the sample, such that the depth relative to the transducer was 65 mm. At that depth, the diffraction limit is 1.17 mm, thus the phalanges are not resolvable. At the 65 mm depth, a set of additional low-resolution reference images was acquired using the following methods: plane wave imaging (Fig. 8c); coherent compounding of five-angled plane waves (Fig. 8d), and a two-way focusing (Fig. 8e).

A multi-foci pattern was designed for a distance of *z = *65 mm, with five foci separated by a distance of 2.2 mm, thus the pattern covered an area of 10.1 mm. The shift between successive patterns equals 0.44 mm. The apodization and delay maps used for the experiments in Figs. 6 and 8 and the Fourier transform of the cross section of the transmitted field at the focal depth is found in Supplementary Fig. 6. ASI imaging was performed similarly to the previous experiments, Fig. 8f–h presents three of the predicted emitted patterns, and the corresponding received images are provided in Fig. 8i–k. The super-resolution image is shown in Fig. 8l. In all of the reference images captured at *z* = 65 mm, the phalanges were not resolvable, whereas they were resolved in the super-resolution image (Fig. 8l) and the underlying structure is exposed. The similarity to the high-resolution image (Fig. 8b) validates the proposed method.

## Discussion

We describe the implementation of a structured illumination method designed to enhance the resolution of ultrasound imaging and open the door to new biomedical and basic science applications. By superimposing time-varying patterns on an object and multiplying each captured image by the corresponding decoding pattern, the effective resolution is improved. The method requires the acquisition of a few images (typically five), to achieve super-resolution similar to SIM in a region of interest. As the phase and apodization maps can change dynamically during transmission, and the post processing associated with the method is simple and straightforward, the entire process can be performed in real-time.

With large element count arrays and programmable ultrasound systems, we now have the potential to optimize biomedical imaging in separate survey and detail operating modes, where a specific set of ultrasonic transmissions is created for regions in which the details are required. Initially, a survey mode would be used to image the object using conventional two-way focusing. At a desired location where fine detail needs to be resolved, the ASI method will be applied. This idea is analogous to the use of methods as color flow imaging and pulsed Doppler^39^. We applied this technique with a relatively low ultrasound frequency of 3.6 MHz, which dictated the lateral resolution; however, applying the technique with a substantially higher frequency will improve the resolution in proportion to the enhancement in frequency. Implementation of these techniques in routine biomedical imaging will be facilitated by two-dimensional transducer arrays that are under development for radiology applications.

In our preliminary experiments, examples of high dynamic range targets (wires and phalanges) were provided to validate the technique. Within this realm of high dynamic range targets are important medical applications, e.g., applying super-resolution to detect changes in musculoskeletal tissues resulting from arthritis and other degenerative processes. Additional work will be required to establish the resolution and image contrast that can be obtained in other organ systems to map small lesions, tumor borders, and layers of peripheral vascular walls. ASI should also be evaluated in ultrasound applications that require high spatial resolution to distinguish anatomical landmarks, such as in cardiac and fetal imaging, and ultrasound-guided interventions, fetal surgeries^40, 41^, and cardiovascular interventions^42^.

We compared ASI to traditional ultrasound imaging protocols and demonstrated enhanced spatial resolution. An additional advantage compared to two-way focusing methods is in the acquisition time. As ASI scans the image with a multi-foci pattern, the reduction in acquisition time is proportional to the number of foci. For the five-foci patterns used in the paper, the ASI method is five times faster than two-way focusing. With respect to the field of view, improving the resolution by a factor of two over one-way focusing requires a small spacing between foci and therefore a small field of view. Scanning a larger field of view can be done by combining images from multiple foci patterns.

The technique demonstrated here has fundamental differences as compared with SIM. In optics, the pattern is projected on top of the sample using an external module. However, in ultrasound, no external module is required, therefore this method can be applied to commercial ultrasound systems without the need for additional components. Another difference stems from the nature of image formation in optics and ultrasound. In optics, the entire image is captured simultaneously, thus SIM, which requires the capture of additional images of the same scene, is by definition time consuming. However, in medical ultrasound, a two-way focused image is often acquired sequentially (one line at a time) and therefore, ASI harnesses this acquisition process for achieving super-resolution. Also, unlike other recently reported super-resolution ultrasound techniques^43–45^ that require the use of microbubble contrast agents and thus are limited only to vascular imaging, the proposed method is designed for general ultrasound imaging^22–24^. In addition, other ultrasound super-resolution methods require thousands of frames to assemble the super-resolution image, whereas the proposed method requires the acquisition of a few frames and thus is applicable for real-time imaging.

In conclusion, it is possible to significantly improve the lateral resolution and frequency transfer function of an ultrasound imaging system using structured illumination. As a result, complex biological samples can be imaged at a substantially higher level of detail.

## Methods

### Sample preparation

Two samples were used for these experiments. The first is a multi-tissue ultrasound phantom (CIRS, Virginia, USA) consisting of a Zerdine^®^ hydrogel polymer, which has tissue mimicking properties^46^. The phantom contains targets with spacing calibrated to quantify the axial and lateral resolution of an ultrasound system. The target set studied here was located at a depth of 29 mm and is composed of 0.8 mm nylon wires, with lateral and axial separation distances of 0.25, 0.5, 1, 2, 3, and 4 mm, as illustrated in Fig. 6a. The speed of sound within this phantom is 1540 m s^−1^. Each target was imaged multiple times and typical results are presented.

### Ex vivo sample preparation

All animal-related work performed by our laboratory was approved by the relevant institutional committees. The ex vivo sample was a Sprague–Dawley rat’s paw embedded within agarose. The paw was located at a depth of 34 mm. Overall 2% agar (Alfa Aesar, MA, USA) was mixed with deionized water at ambient temperature and heated until all powder was dissolved. A layer of the degassed solution was poured into a mold and cooled until congealed. The rat’s paw was placed on top of this layer, and covered by the remainder of the solution and allowed to congeal. The speed of sound within this sample was ~1500 m s^−1^ ^46^.

### Computation and ultrasound imaging

The design of the patterns and the post processing of the images were implemented in MATLAB (version 2016b, MathWorks, Natick, MA, USA). The acoustic pressure field corresponding to the calculated phase and apodization maps was simulated using Field II software^47^. Both programs run on a Dell OptiPlex 7040 PC with a Windows 10 Enterprise 64-bit operating system, Intel^®^ Core™ i7-6700 processor, 3.40 GHz, 16 GB RAM. Ultrasound imaging was performed using the Verasonics ultrasound system (Vantage 256, Verasonics Inc., Redmond, WA, USA), at a center frequency of 3.6 MHz (lambda of 0.427 mm) and with a phased array sector transducer P6-3 (ATL Ultrasound Inc., Bothell, WA, USA). The excitation for each transmitted pulse was ~1 cycle. The transducer has 128 elements, with an element size of 0.22 mm and therefore a total aperture of ~28.2 mm. The transducer was fixed to an optical plate throughout each experiment. For each experiment, three low-resolution images of the same field of view were acquired with the same imaging parameters and used as reference images to compare to the ASI method. These reference images included: an image resulting from a plane wave transmission (with parallel receive beamforming); an image coherently compounded from five-angled plane waves (coherent plane wave compounding is a method that is frequently used to improve image quality in ultrasound. For a depth of 30 mm, the angles were −30°, −15°, 0, 15°, 30°, and for depth of 65 mm, the angles were −15°, −7.5°, 0, 7.5°, 15° as in ref. ^33^); and an image resulting from two-way focusing. For each depth the focus was set to be at the object’s position. At a depth of 30 mm, imaging was performed with 128 lines, with a line width of 0.2 mm. At a depth of 65 mm, 256 lines were acquired with the same line width.

For pattern generation, a single plane wave imaging mode was chosen. For the super-resolution reconstruction process, a precise estimate of the patterns generated is essential. Visualization of the transmitted pattern was facilitated using the Verasonics built in Matlab “ShowTXPD” function. This function provides a 2D color encoded display of the transmit beam for every pixel in the ultrasound image field of view, and accounts for the transducer geometry, the medium sound speed and attenuation, as well as all transmit apodization and waveform parameters. The imaged pressure field, as well as the estimated emitted field were saved and post processed to generate the super-resolution image.

### ASI method

The ASI method requires the following steps: determine the depth to be imaged; determine the shape of the desired pattern at the focal depth (number of foci and the spacing between them); calculate the transducer phase and apodization maps using the Gerchberg–Saxton algorithm; simulate the emitted fields to be used in the post processing; excite the transducer with the apodization maps and record the received data; multiply each of the five images by the corresponding simulated field; sum and average the five decoded images; and finally subtract the average of the five ASI images (without the decoding process) from the averaged decoded images to achieve the final high-resolution image.

### Hydrophone measurements

The acoustic pressure field was measured in a degassed water tank using a wide-band needle hydrophone (HNP-0400, Onda, Sunnyvale, CA, USA) with an active aperture of 0.4 mm. The hydrophone probe was mounted on a three-dimensional positioning system (Newport motion controller ESP 300, Newport 443 series. The pressure signals received by the hydrophone were first displayed on a digital oscilloscope (DPO4034, Tektronix, OR, USA), and further recorded and converted via post processing to a normalized pressure map.

### Data availability

The data sets generated during and/or analyzed during the current study are available from the corresponding author on reasonable request.

## Electronic supplementary material

Supplementary Information
