# Supplementary material for: Acoustical structured illumination for super-resolution ultrasound imaging
Source: Commun Biol. 2018 Jan 22;1:3. doi: 10.1038/s42003-017-0003-5 (PMC5988254; doi:10.1038/s42003-017-0003-5)
Supplement: Supplementary file 1 — Supplementary Information [file 42003_2017_3_MOESM1_ESM.pdf]

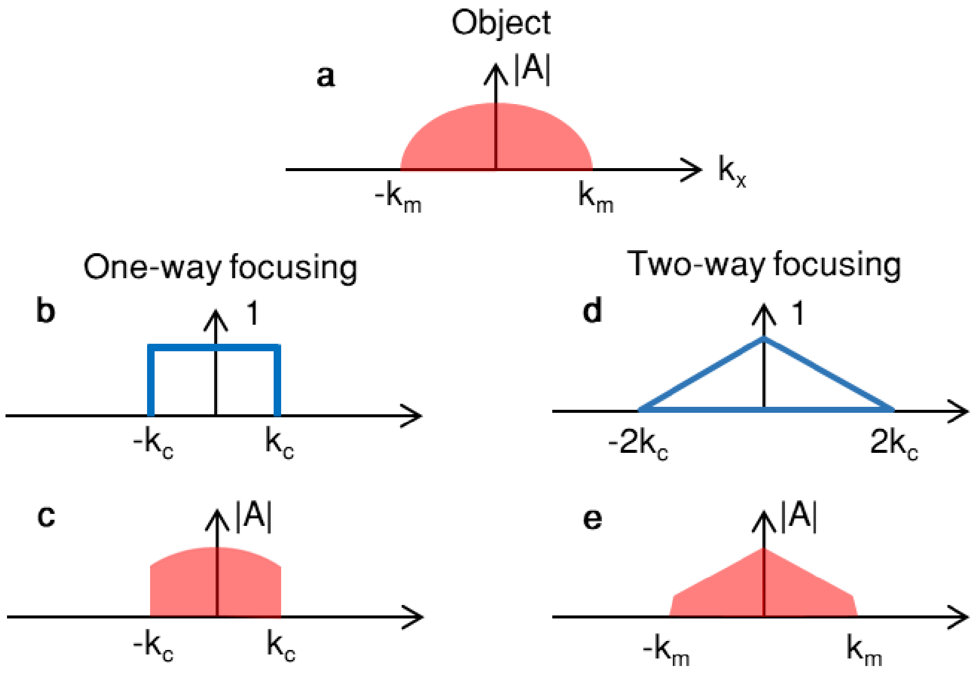

Supplementary Fig. 1 K-space representation of one-way and two-way focusing. a, The imaged object has spectral components from -km to km. **b,** For one-way focusing (such as plane wave transmission with synthetic focusing on receive), the lateral k-space transfer function is rectangular in shape with a cutoff frequency of kc. **c,** The Fourier transform of the received image for plane wave imaging is the multiplication of the Fourier transform of the object and the rectangular k-space transfer function. The result is low-pass filtering of the object. **d,** The transfer function for two-way beam formation is equal to the convolution between the transfer function on transmit and receive. Assuming both are rectangles with the same width, the transmit-receive transfer function is a triangle with twice the cutoff frequency compared to transmission alone. **e,** The Fourier transform of the received image for two-way focusing is the multiplication of the Fourier transform of the object and the triangular transfer function, resulting in attenuation of high frequencies.

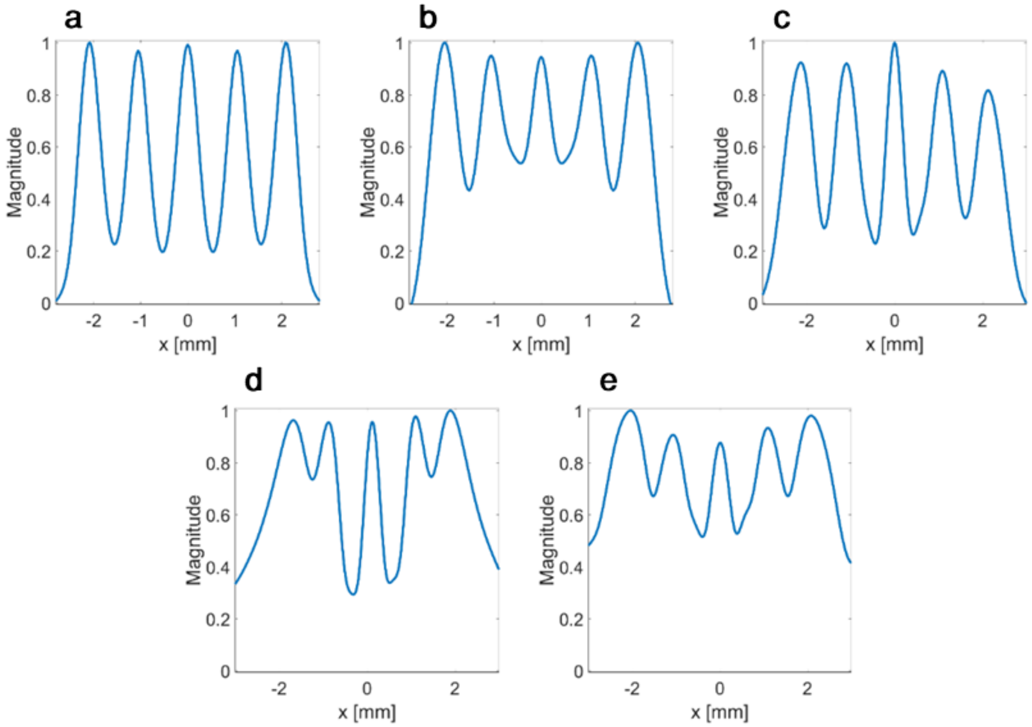

Supplementary Fig. 2 Line profiles acquired at the focal depth (30 mm) of the emitted fields presented in Fig. 4. a, Simulated emitted ultrasound field for continuous wave (CW) transmission, using the modified Gerchberg–Saxton algorithm for Δd=1.1 mm (0.5/k_c). **b,** Simulated emitted ultrasound field for 1 cycle transmission, using the modified Gerchberg–Saxton algorithm for Δd=1.1 mm. **c**, Experimental hydrophone measurement of the pattern presented in **b**. **d,** Simulated emitted field for 1 cycle transmission using the multiline transmit (MLT) method for Δd=1.1 mm. **e,** Experimental hydrophone measurement of the pattern presented in **d**.

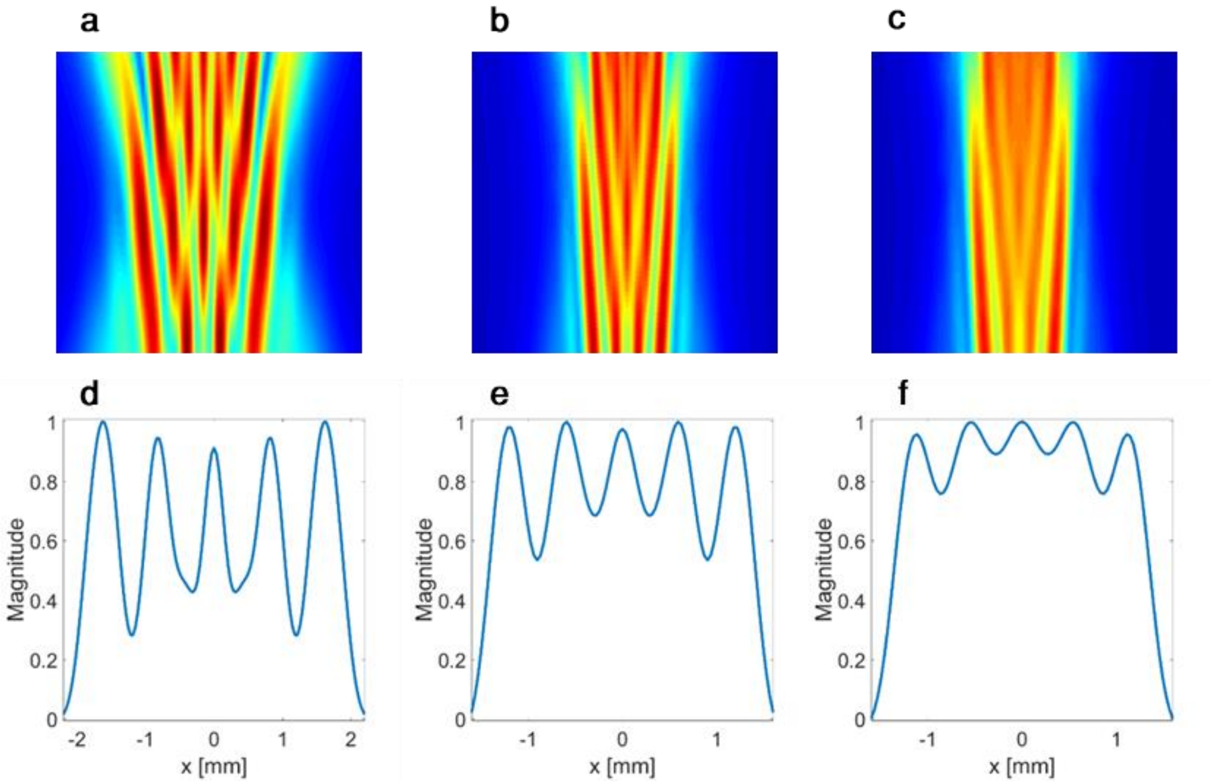

Supplementary Fig. 3 The influence of foci spacing on the generated pattern. a-c, Simulated emitted ultrasound field for 1 cycle transmission, using the modified Gerchberg–Saxton algorithm for Δd= 0.8 ($0.7/k_c$), 0.58 ($0.97/k_c$) and 0.55 ($1/k_c$) mm, respectively. Results are normalized with axes and colorbar identical to Fig. 4. **d-f,** Line profiles acquired at the focal depth of the emitted fields presented in **a-c** respectively.

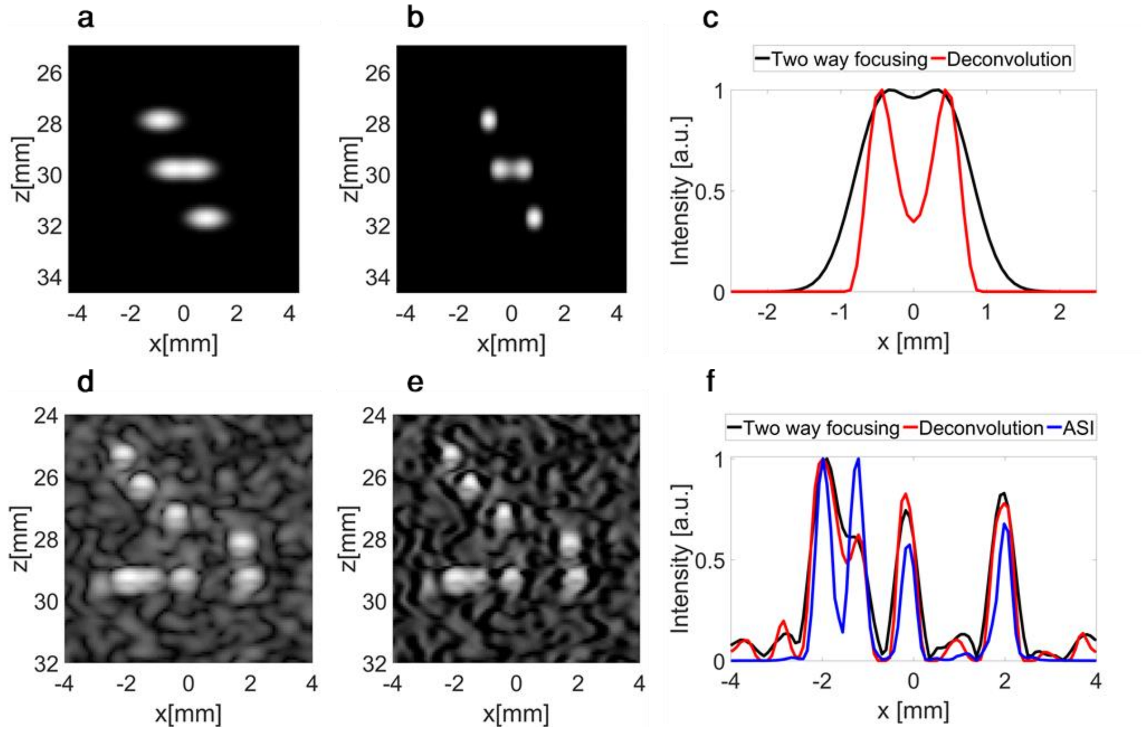

Supplementary Fig. 4. Comparison to ultrasound blind deconvolution. Images are presented with a dynamic range of 25 dB. **a-c,** Simulation results of 5 wires. **a,** Two-way focused image of the wires. **b,** Result of ultrasound deconvolution on **a**. **c,** Cross section of **a** and **b** along the two closely spaced wires (z=30 mm). **d,** Two-way focused image of the lateral resolution target presented in Fig 6d. **e,** Result of ultrasound deconvolution on **d**. **f,** Cross section along the lateral resolution target (z = 29.3 mm), for **d,e** and the ASI result from Fig. 6o.

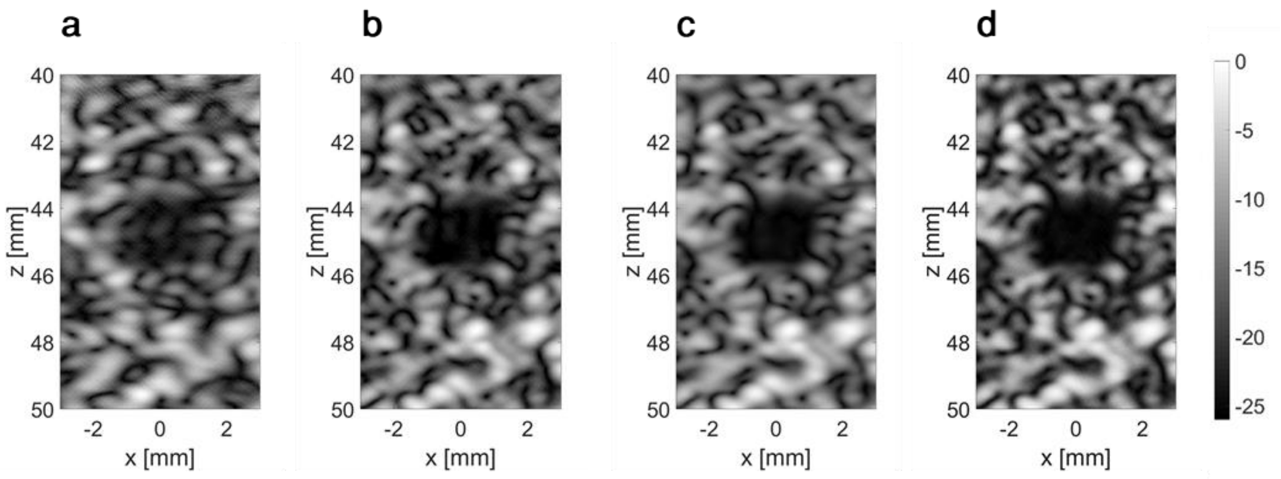

Supplementary Fig. 5. Images of a cyst in a homogeneous multipurpose ultrasound imaging phantom. Images with a dynamic range of 25 dB are displayed for an image acquired with: **a,** one-way focusing. **b,** coherent compounding of 5-angled plane waves. **c,** two-way focusing, and **d,** ASI imaging.

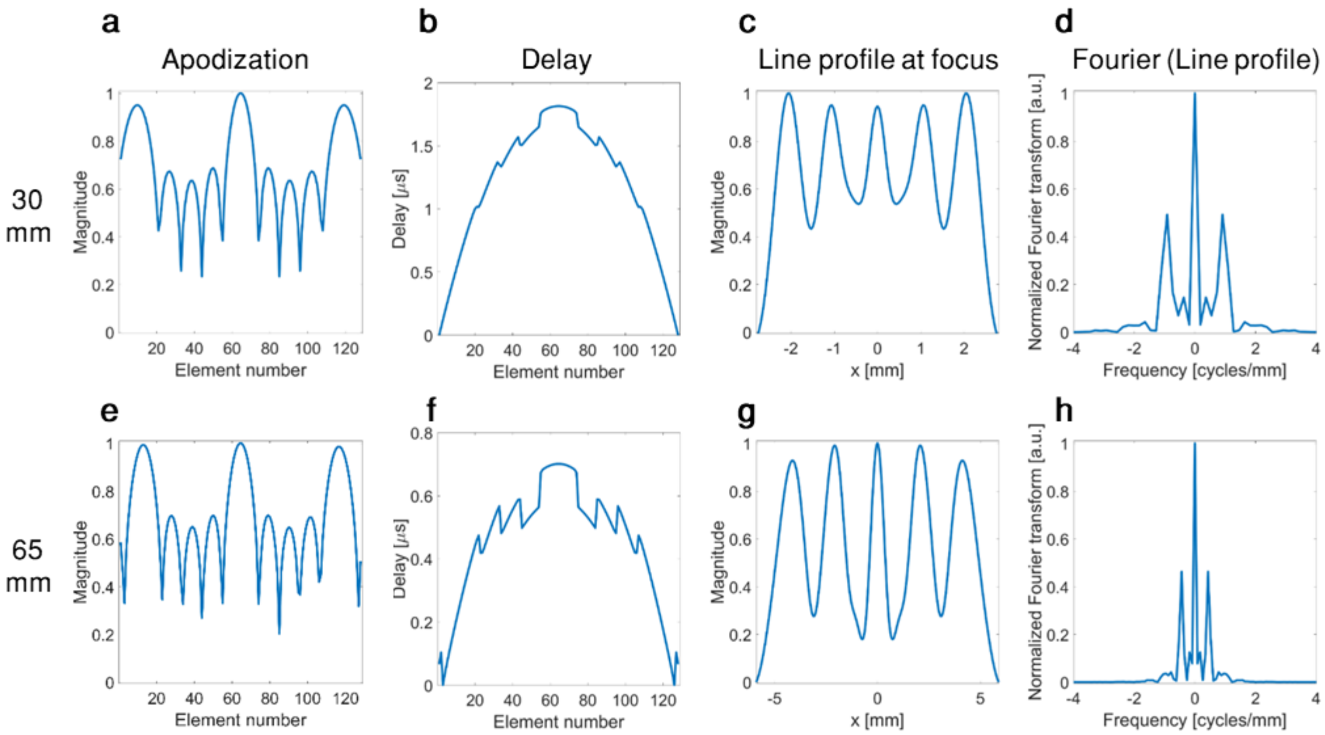

Supplementary Fig. 6 Transducer apodization and delay profiles and the generated patterns. Upper and lower rows represent depths of 30 and 65 mm respectively. **a,e,** apodization profiles. **b,f,** transducer delay profile. (c),(g) line profile of the generated pattern at the focal depth. (d),(h) Fourier transform of (c),(g) respectively.

	Contrast (dB)	CNR (dB)	Speckle cell size (mm)
**PW**	-14.12	5.33	0.91
**5 angles**	-18.53	6.7	0.79
**Two way focusing**	-20.9	7.46	0.76
**ASI**	-20.56	7.25	0.52

Supplementary Table 1. Calculation of the contrast, contrast to noise ratio (CNR), and speckle resolution cell size were for a anechoic cyst target using the four ultrasound imaging methods presented in Supplementary Fig. 5.

Supplementary Note 1: Mathematical framework for ASI

First, let us consider the case of plane wave imaging that is focused only on receive to form the plane wave image *(PW(x))*. On receive, the image is convolved with the point spread function of the focused transducer.

$$PW(x) = \int_{-\infty}^{\infty} Obj(x')PSF(x - x')dx' \quad (1)$$

where *Obj* is the object to be imaged and *PSF* is the point spread function for the focused transducer. The k-space lateral representation for one-way focusing is the Fourier transform of the point spread function. For a rectangle aperture and a monochromatic wave, this k-space representation is given by a rectangle function: $rect(\frac{k_x}{k_c})$, where k_x is the lateral spatial frequency and k_c is the cutoff frequency of the system that is equal to the inverse of the lateral diffraction limit, as defined according to Equation 1.

In ASI, a periodic pattern or grating is superimposed on the object. The Gerchberg–Saxton algorithm is used to determine the transducer phase and apodization maps that generate the desired encoding pattern at the focal depth. In our design for single cycle transmission, the desired grating requested for the Gerchberg–Saxton algorithm in the spatial domain is a sum of delta functions spaced by a distance of Δd and in k-space, is also the sum of delta functions. The algorithm forward and backward propagates the field between the transducer and focal planes, and thus the result is diffraction limited and low pass filtered with the transducer's cutoff frequency k_c. If the requested set of delta functions is such that only 3 delta components ($-k_1$, 0, $+k_1$) are below k_c, the k-space representation of the pattern generated at the focal zone is given by (Fig. 2b):

$$\tilde{g}_1(k_x) = C \cdot \sum_{n=-1}^{1} \delta(k_x - nk_1) \quad (2)$$

where $\tilde{g}_1$ is the Fourier transform of the generated grating g_1. In the spatial domain, the grating is a raised cosine function:

$$g_1(x) = 1 + \frac{1}{2}\cos(2\pi x k_1). \quad (3)$$

During transmission, this encoding grating is generated at the object's position, yielding a spatial multiplication of the two, or a frequency mixing that generates duplications of the k-space representation of the object, as in Fig. 2c. The grating is then shifted laterally between successive transmitted pulses, creating an increasing phase term (ϕ) for each transmission.

On receive, one-way focusing is applied to the beamformed echoes and thus the received data are lowpass filtered with a cutoff frequency of k_c to yield Fig. 2d. In the spatial domain, the result (termed *Receive(x,*ϕ*)*) is therefore:

$$Recieve(x,\phi) = \int_{-\infty}^{\infty} Obj(x')g_1(x' - \phi)PSF(x - x')dx' \quad (4)$$

The set of captured images is post processed where each image is multiplied by a second grating g_2, and summed over a single period of the pattern Δd to form the reconstructed image *R(x)*:

$$R(x) = \frac{1}{\Delta d}\int_{0}^{\Delta d} \int_{-\infty}^{\infty} Obj(x')g_1(x' - \phi)p(x - x')dx' \, g_2(x - \phi)d\phi \quad (5)$$

Since the object is assumed to be stationary during the scan, the integration order can be reversed, and integration over the shifted patterns performed first:

$$\alpha(x,x') = \frac{1}{\Delta d}\int_0^{\Delta d} g_1(x'-\phi)g_2(x-\phi)d\phi \tag{6}$$

g_1 can be written explicitly:

$$g_1(x'-\phi) = \frac{1}{2}+\frac{1}{2}\cos[2\pi(x'-\phi)k_1] = \frac{1}{2}+\frac{1}{4}e^{2\pi i(x'-\phi)k_1}+\frac{1}{4}e^{-2\pi i(x'-\phi)k_1} \tag{7}$$

Since g_2, which is the decoding grating, is added digitally, it can be chosen to be:

$$g_2(x) = 2 + 8\cos(2\pi x k_1) \tag{8}$$

g_2 is also shifted between subsequent pulses with the same phase term ϕ:

$$g_2(x-\phi) = 2 + 8\cos[2\pi(x-\phi)k_1] = 2 + 4e^{2\pi i(x-\phi)k_1} + 4e^{-2\pi i(x-\phi)k_1} \tag{9}$$

The integral over the shifted patterns presented in Supplementary Equation 6 can be derived as:

$$\alpha(x,x') = \frac{1}{\Delta d}\int_0^{\Delta d} g_1(x'-\phi)g_2(x-\phi)d\phi$$

$$= \frac{1}{\Delta d}\int_0^{\Delta d} \left[\frac{1}{2}+\frac{1}{4}e^{2\pi i(x-\phi)k_1}+\frac{1}{4}e^{-2\pi i(x-\phi)k_1}\right]\left[2+4e^{2\pi i(x-\phi)k_1}+4e^{-2\pi i(x-\phi)k_1}\right]d\phi$$

$$=$$

$$\text{I)}\quad \frac{1}{\Delta d}\int_0^{\Delta d} d\phi = 1$$

$$\text{II)}\quad \frac{1}{\Delta d}\int_0^{\Delta d} 2e^{2\pi i(x-\phi)k_1}d\phi = \frac{2}{\Delta d}e^{2\pi i x k_1}\int_0^{\Delta d} e^{-2\pi i\phi k_1}d\phi = 0$$

$$\text{III)}\quad \frac{1}{\Delta d}\int_0^{\Delta d} 2e^{-2\pi i(x-\phi)k_1}d\phi = \frac{2}{\Delta d}e^{-2\pi i x k_1}\int_0^{\Delta d} e^{2\pi i\phi k_1}d\phi = 0 \tag{10}$$

$$\text{IV)}\quad \frac{1}{\Delta d}\int_0^{\Delta d} \frac{1}{2}e^{2\pi i(x-\phi)k_1}d\phi = \frac{1}{2\Delta d}e^{2\pi i x' k_1}\int_0^{\Delta d} e^{-2\pi i\phi k_1}d\phi = 0$$

$$\text{V)}\quad \frac{1}{\Delta d}\int_0^{\Delta d} e^{2\pi i(x'+x-2\phi)k_1}d\phi = \frac{1}{\Delta d}e^{2\pi i(x'+x)k_1}\int_0^{\Delta d} e^{-4\pi i\phi k_1}d\phi = 0$$

$$\text{VI)}\quad \frac{1}{\Delta d}\int_0^{\Delta d} e^{2\pi i(x'-x)k_1}d\phi = \frac{1}{\Delta d}e^{2\pi i(x'-x)k_1}\int_0^{\Delta d} d\phi = e^{2\pi i(x'-x)k_1}$$

$$\text{VII)}\quad \frac{1}{\Delta d}\int_0^{\Delta d} e^{-2\pi i(x'-\phi)k_1}d\phi = \frac{1}{\Delta d}e^{-2\pi i x' k_1}\int_0^{\Delta d} e^{2\pi i\phi k_1}d\phi = 0$$

VIII) $\frac{1}{\Delta d}\int_0^{\Delta d} e^{-2\pi i(x'-x)k_1} d\phi = \frac{1}{\Delta d} e^{-2\pi i(x'-x)k_1}\int_0^{\Delta d} d\phi = e^{-2\pi i(x'-x)k_1}$

IX) $\frac{1}{\Delta d}\int_0^{\Delta d} e^{-2\pi i(x'+x-2\phi)k_1} d\phi = \frac{1}{\Delta d} e^{-2\pi i(x'+x)k_1}\int_0^{\Delta d} e^{+4\pi i\phi k_1} d\phi = 0$

$$\alpha(x,x') = 1 + e^{2\pi i(x'-x)k_1} + e^{-2\pi i(x'-x)k_1}$$

Placing it back into Supplementary Equation 5:

$$R(x) = \int_{-\infty}^{\infty} Obj(x')PSF(x-x')\left[1 + e^{2\pi i(x'-x)k_1} + e^{-2\pi i(x'-x)k_1}\right]dx' \quad (11)$$

which can be rewritten as:

$$R(x) = Obj(x) * \left[PSF(x) + PSF(x)\cdot e^{2\pi i x k_1} + PSF(x)\cdot e^{-2\pi i x k_1}\right] \quad (12)$$

In k-space:

$$\tilde{R}(k_x) = \widetilde{Obj}(k_x)\cdot\left[\widetilde{PSF}(k_x) + \widetilde{PSF}(k_x+k_1) + \widetilde{PSF}(k_x-k_1)\right] \quad (13)$$

where $\widetilde{Obj}$ and $\widetilde{PSF}$ represent the Fourier transform of the object and the point spread function respectively. Since $\widetilde{PSF}$ is the rectangular lateral transfer function: $rect(\frac{k_x}{k_c})$, the result contains two additional duplications of the transfer function centered at $-k_1$ and $+k_1$ together with the original transfer function centered at 0. The result is thus the sum of three rectangles with a cutoff frequency of (k_c+k_1) (Fig. 2g). In order to achieve a flat effective transfer function, a low resolution image (*LR(x)*) is subtracted from $R(x)$, which yields the super-resolution image (*SR(x)*) (Fig. 2f). The low-resolution image is an average of the five captured images of ASI (without the additional post processing):

$$SR(x) = R(x) - LR(x) \quad (14)$$

The effective transfer function (ASI transfer function) for the ASI method (as in Fig. 2g) is therefore:

$$ASI\ transfer\ function(k_x) = \widetilde{PSF}(k_x+k_1) + \widetilde{PSF}(k_x-k_1) \quad (15)$$

Since the transmitted pattern is limited by the cutoff frequency k_c, the maximal enhancement in resolution occurs when k_1=k_c and yields a resolution improvement by a factor of 2 compared to plane wave imaging.

Supplementary Note 2: Cyst brightness parameters

Contrast is a measure of the difference in brightness between the cyst and the surrounding region.

$$Contrast = \frac{\mu_o - \mu_i}{\mu_o} \tag{16}$$

where μ_o is the mean of a speckle region outside the cyst, and μ_i is the mean of a region inside the cyst. Since the cyst signal is lower than the surrounding speckle, the contrast is typically presented as a negative value in decibels:

$$Contrast(dB) = 20\log_{10}(1 - Contrast) = 20\log_{10}\left(\frac{\mu_i}{\mu_o}\right) \tag{17}$$

The CNR is a parameter that indicates the detectability of a lesion compared to the surrounding background.

$$CNR(dB) = 20\log_{10}\left(\frac{\mu_o - \mu_i}{\sqrt{{\sigma_o}^2 + {\sigma_i}^2}}\right) \tag{18}$$

where σ_o is the standard deviation of a speckle region outside the cyst, and σ_i is the standard deviation of a region inside the cyst.
